# Construction of SNP Fingerprinting and Genetic Diversity Analysis of Eggplant Based on KASP Technology

**DOI:** 10.3390/ijms26115312

**Published:** 2025-05-31

**Authors:** Wuhong Wang, Hongtao Pang, Na Hu, Haijiao Hu, Tianhua Hu, Yaqin Yan, Jinglei Wang, Jiaqi Ai, Chonglai Bao, Qingzhen Wei

**Affiliations:** Institute of Vegetable, Zhejiang Academy of Agricultural Science, Hangzhou 310021, China; wangwh@zaas.ac.cn (W.W.); panghongtao@stu.zafu.edu.cn (H.P.); huna01@stu.zafu.edu.cn (N.H.); huhj@zaas.ac.cn (H.H.); huth@zaas.ac.cn (T.H.); yanyq@zaas.ac.cn (Y.Y.); wangjinglei@zaas.ac.cn (J.W.); ajq@stu.zafu.edu.cn (J.A.)

**Keywords:** eggplant, KASP, DNA fingerprinting, genetic diversity, population structure

## Abstract

Eggplant (*Solanum melongena*) is a significant vegetable in the Solanaceae family. Significant progress has been made in genetic diversity analysis and fingerprinting construction for crops such as tomatoes and peppers within the same family, but research on eggplants in these aspects remains relatively limited. Current germplasm identification using fingerprinting primarily relies on traditional SSR markers, which suffer from limited polymorphism and labor-intensive workflows. This study aimed to identify high-quality single nucleotide polymorphisms (SNPs), develop reliable Kompetitive Allele-Specific PCR (KASP) markers for eggplant genotyping, and then conduct fingerprint construction and genetic diversity analysis. The ultimate goals were to achieve a precise identification of eggplant varieties and deeply explore the genetic background and evolutionary patterns of eggplant germplasm. In this study, 49 representative eggplant accessions were re-sequenced. After data quality control, sequence alignment, and multiple rounds of screening, 224 high-quality SNPs were identified. Based on these SNPs, 96 SNPs were selected to develop KASP markers. These markers can provide abundant genetic markers for eggplant genetic research, which are used to deeply explore the genetic background and conduct genetic diversity analysis. After multiple rounds of rigorous verification, 32 core candidate markers were finally screened out. The average polymorphic information content (PIC) and gene diversity (GD) values were 0.36 and 0.46, respectively. Phylogenetic tree, population structure, and principal component analyses divided the 280 eggplant accessions into eight distinct groups. Through the analysis of minimal core markers and core germplasm, 23 core SNP markers and a subset of 56 core germplasm accessions were identified, leading to the establishment of a comprehensive fingerprinting system for all 280 accessions. Our findings provide a foundational genetic resource for eggplant germplasm identification and offer significant support for future breeding efforts.

## 1. Introduction

Eggplant (*Solanum melongena* L., 2n = 24) is an economically important horticultural crop in the Solanum family [1]. It can be broadly categorized into Eastern and Western groups in Southeast Asia [2]. Eggplant production is now dominated by China, which produced over 38.31 million tons cultivated across 0.82 million hectares (FAO, 2022). Eggplant holds extremely high economic value and is rich in minerals and vitamins. It plays a positive role in preventing and managing various health issues, including cancer, diabetes, hyperlipidemia, high cholesterol, atherosclerosis, anti-angiogenesis, and inflammation [3,4,5]. China is extremely rich in eggplant (*Solanum melongena*) germplasm resources and currently holds the largest collection of such resources in the world [6]. Eggplant germplasm resources are the carriers of eggplant genetic information and an important component of eggplant genetic diversity. They not only play a significant role in maintaining the stability of the agricultural system but also serve as the material basis for eggplant variety breeding, representing a precious resource for humanity [7]. Genetic diversity refers to the total genetic variation within a species, and the greater the degree of variation, the stronger the adaptability to the environment. Therefore, the richness of genetic diversity can represent the competitive ability of such seed resources in nature. The study of genetic diversity in germplasm resources, understanding the closeness of the genetic relationship and the degree of variation among various varieties in germplasm resources, enables us to better explore and utilize genotype resources based on these genetic relationships and variations in the process of new variety breeding, laying a theoretical foundation for the selection and improvement of new varieties [8]. Under the influence of climate extremes, environmental pollution, and population growth, the resistance and yield of crops emerged as a paramount concern in crop breeding [9,10]. Concurrently, the enhancement of consumer awareness and economic status has resulted in an intensified emphasis on nutritious and organic food [11]. This trend has witnessed a growing number of consumers placing greater emphasis on their health and allocating more resources towards the acquisition of nutritious organic foods [12]. Consequently, breeders are shifting their focus from traditional agronomic traits such as yield and disease resistance to prioritize flavor and nutritional profiles. The incorporation of diverse germplasm is essential for the successful cultivation of new varieties. This practice not only enriches genetic diversity and expands the varietal gene pool but also strengthens the adaptability of crops across diverse environmental niches, thereby facilitating the creation of novel cultivars with superior adaptive capabilities. Moreover, it addresses the multifaceted demands of agricultural product markets, enhances the market competitiveness of agricultural products, and explores and penetrates new market segments. However, the issue of misnaming, where different species are labeled with the same name or a single species is identified by various names, is inevitably present. This issue encompasses commercial varieties and presents a substantial menace to the interests of both breeders and agricultural producers. Therefore, the development of a convenient, efficient, and precise method for the identification of eggplant varieties is imperative. Such a method would facilitate the conservation and exploitation of eggplant genetic diversity, thereby aiding in the advancement and prosperity of the eggplant breeding industry.

The development of molecular marker technologies, which are tools used to identify and differentiate biological individuals or populations by detecting polymorphisms in DNA sequences, has provided powerful resources for research in plant genetics and breeding. Early molecular marker techniques, such as Restriction Fragment Length Polymorphism (RFLP), were initially employed in the 1980s to detect variations in DNA sequences by exploiting polymorphisms within restriction enzyme sites [13,14]. Consequently, Simple Sequence Repeat (SSR) markers gained extensive usage due to their high polymorphism and codominance [15]. Random Amplified Polymorphic DNA (RAPD) markers, characterized by the use of random primers to amplify DNA fragments, became popular for their simplicity [16]. Amplified Fragment Length Polymorphism (AFLP) combined the advantages of RFLP and PCR, facilitating the generation of numerous markers without requiring prior sequence information [17]. With the advancement of technology, Inter-Simple Sequence Repeat (ISSR) markers, which exploit variations in microsatellite amplification, have provided enhanced resolution [18]. SNP markers, widely distributed and genetically stable, have become one of the most widely used molecular markers in recent years [19]. The emergence of Sequence-Related Amplified Polymorphism (SRAP), Target Region Amplified Polymorphism (TRAP), and Start Codon Targeted (SCoT) markers has provided more direct insights into gene function by, respectively, amplifying open reading frame regions and promoter regions [20,21,22]. The continuous evolution of molecular marker technologies has significantly advanced research in genetic mapping, gene localization, and germplasm identification.

The construction of DNA fingerprinting is an important application field of molecular marker technology, providing precise methods for plant variety identification, germplasm conservation, and genetic diversity studies. DNA fingerprinting databases have been implemented for various plant species such as rice [23], maize [24], potato [25], sweet potato [26], cowpea [27], *Leymus chinensis* [28], and passionfruit [29]. SNP markers, characterized by their large numbers, widespread distribution, high genetic stability, and strong heritability, are highly suitable for high-throughput detection. These attributes render them invaluable in the construction of DNA fingerprinting profiles and have facilitated their widespread application in genomics research. In Broccoli (*Brassica oleracea* var. *italica*), Shen et al. [30]. sequenced 23 accessions, identified 100 SNPs for KASP assays, and selected 28 core markers for fingerprinting. The study revealed that 372 accessions clustered into two major groups with low genetic diversity and a narrow genetic background. Yang employed 41 polymorphic KASP markers to classify 329 cauliflower varieties into 3 groups, creating unique fingerprints and genetic information barcodes for each sample [31]. The genotyping of 27 local Cretan tomato varieties and 2 hybrids was conducted using 11 SSR markers, resulting in the development of fingerprints to facilitate the conservation and utilization of indigenous germplasm [32]. Researchers in Pakistan developed DNA fingerprinting and analyzed genetic diversity in 13 new local varieties, identifying 199 SSR markers with 1314 allele pairs and selecting 50 core markers to support tomato breeding [33]. Similarly, population genetic analysis of 300 cucumber accessions revealed 4 subpopulations corresponding to Asia, Europe, America, and Africa. Notably, 293 accessions were distinguished using 82 SNPs, aiding cucumber breeding efforts [34]. The development of fingerprinting technology based on DNA molecular markers has become an important direction in the research of vegetable germplasm resources and molecular breeding. Compared with crops such as tomato and chili in the same family, the research foundation for eggplant is still relatively weak, and its molecular genetic breeding started relatively late. The research on the construction of fingerprinting maps for eggplant is also relatively scarce. In this study, the resequencing data of 49 representative eggplant accessions were used to identify high-quality SNPs. A total of 96 KASP markers were developed for genotyping 286 collected materials, resulting in 32 candidate core markers and 280 well-typed materials for fingerprint construction. The analysis of minimal core markers resulted in the identification of 23 core markers, which were employed for constructing the fingerprint of 280 eggplant accessions. Additionally, a core germplasm was developed using 32 selected candidate markers. This research holds significant practical implications for eggplant breeding. It provides reliable molecular markers for the identification of eggplant varieties, which can facilitate the collection, conservation, and utilization of eggplant germplasm resources.

## 2. Results

### 2.1. Identification of High-Quality SNPs

**Figure 1:**
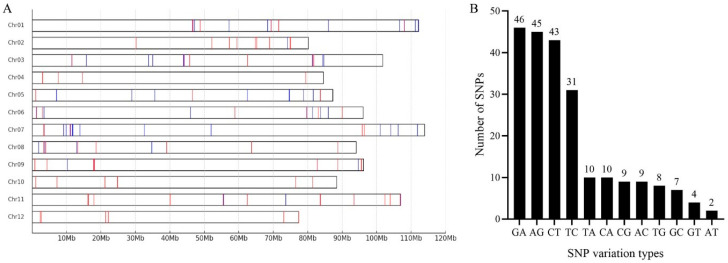
Distribution of 224 high-quality SNPs and genotype statistics. (**A**) The red lines indicate the development site of high-quality SNP markers and the blue lines indicate the development site of KASP markers. (**B**) Statistics of 12 SNP variation types.

The resequencing data from 45 representative eggplant accessions and 4 published eggplant resequencing data were used for SNP selection. A total of 248.48 Gb of raw data were obtained from the resequencing analysis of 45 eggplants, the average Q30 was 92.57%, and the GC% was between 36.76% and 37.96%. After quality filtering and adapter trimming, 244.53 Gb of clean data were obtained for further analyzing. The statistics of sequencing data quality are presented in Supplementary Table S1. A total of 4,869,502 SNPs were identified from the resequencing data of 49 eggplant accessions by aligning with the HQ1315 reference genome. By employing Python scripts, GATK, and vcftools, we filtered SNPs to ensure no other SNPs were present within 200 bp upstream or downstream of each position. The missing rate, allele frequency, and diversity of SNPs were computed to assess the role of these SNPs in evaluating genetic relationships and diversity among 286 samples. A total of 224 high-quality SNPs were identified, with minor allele frequencies ranging from 30% to 70%, and these SNPs were evenly distributed across all 12 chromosomes. The 224 SNPs underwent a statistical analysis to investigate the various types of variations, leading to the identification of 12 distinct variation categories. Among them, A/G (20%), C/T (19%), T/C (19%), and G/A (13%) exhibited the highest proportion of genotypes among all variation types (Figure 1B). These diverse SNP variation types and their proportions reflect the natural diversity of single-nucleotide polymorphisms in the genome. Among the SNP variant base types, the proportion of conversion types such as the A/G and C/T is much higher than that of the inverted type, which conforms to the general law of the genome and indicates that the eggplant genome has rich genetic variations. Regarding base variations, current studies have achieved A-to-G and C-to-T/G/A editing in plants, thereby achieving the research goals of plant gene function studies and crop variety improvement. The differences in various variation types can effectively reveal the domestication history, and natural selection and artificial selection play a crucial role in the retention or elimination of different base variation types. For example, natural selection reflects the adaptability of the species to the environment through the proportion of base variations, and artificial selection also leads to base differences by intervening in biological evolution. Through these selections, an effective theoretical basis can be provided for molecular breeding and genetic protection. The abundance of these specific genotypes indicates a substantial genetic variation in the eggplant genome, which serves as a valuable resource for genetic studies and breeding programs. The identification of these high-frequency variations is essential as it establishes a fundamental basis for further genetic research and their potential association with important agronomic traits. The reliability of the 224 high-quality SNPs identified in this study further emphasizes their robustness as core markers. Core markers play an indispensable role in genetic research, providing stable and consistent genetic landmarks which can be universally applied across diverse studies and populations. The exceptional quality and extensive range of the variation types exhibited by these SNPs indicate their suitability for applications such as genetic mapping, marker-assisted selection, and other endeavors aimed at comprehending and enhancing the eggplant genome.

### 2.2. Development and Genotyping of KASP

From a pool of 224 high-quality SNPs, we selected 96 SNPs based on an even chromosomal distribution for the development of KASP markers. These markers were then validated and screened across 44 representative samples (production area, peel color, fruit shape, etc.), with the 4 common typing results depicted in Supplementary Figure S1. After a thorough evaluation, we excluded 8 markers due to a high rate of missing data and 46 monomorphic markers. The remaining 42 markers underwent further validation for reliability in a larger set of 286 samples. (In the KASP typing validation, the results of 6 samples were extremely poor. Therefore, these samples were excluded from this study. Moving forward, all analyses will be conducted using the 280 samples after excluding the mentioned samples). The results revealed that three markers exhibited high missing data rates, five were found to be monomorphic, and two exhibited both high missing data rates and monomorphism. After this strict process, 32 KASP markers with good genotyping results were identified. Its reliability was first verified through polymorphism information content (PIC) analysis; the average PIC value of 32 candidate labels in 280 materials was 0.355 (at a moderately high level), indicating that the label quality met the standards. The stability of the markers was further confirmed through genetic diversity analysis (Supplementary Tables S2 and S3), which is applicable to subsequent studies.

### 2.3. Analysis of Genetic Diversity

**Figure 2:**
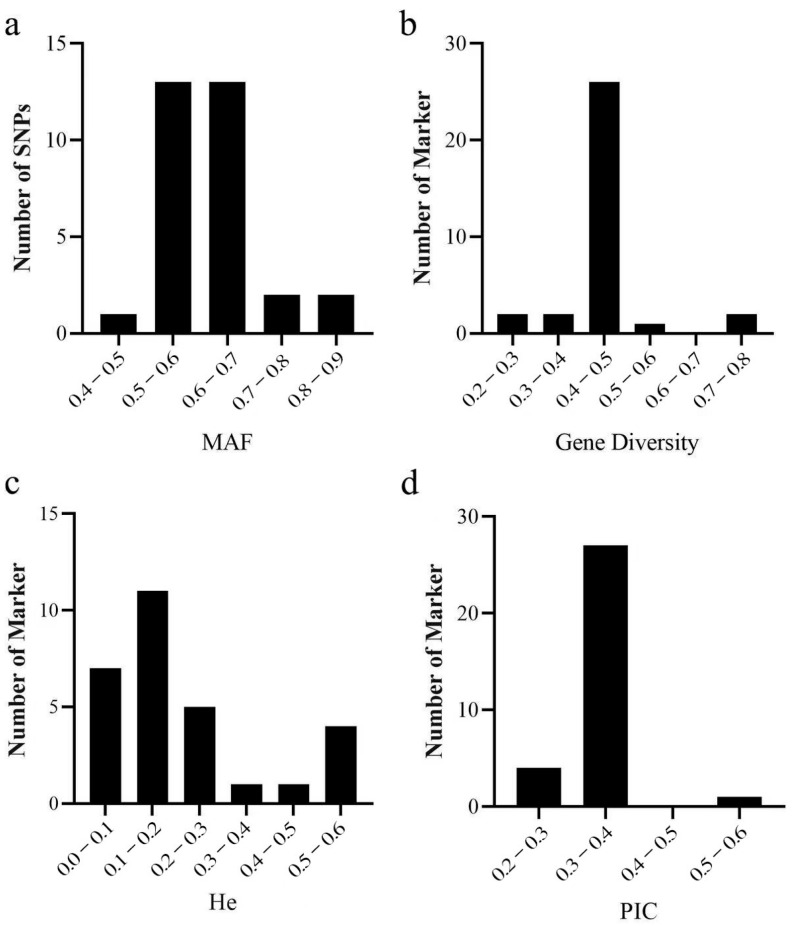
Genetics analysis of 32 candidate core markers in 280 eggplant materials. (**a**) Minor allele frequency (MAF). (**b**) Gene diversity (GD). (**c**) Heterozygosity (He). (**d**) Polymorphic information content (PIC).

The efficacy of KASP genotyping of 280 eggplant materials for the candidate core markers was assessed by evaluating genetic diversity using PowerMarker V3.25 software (Supplementary Table S4). The 32 KASP markers exhibited Minor Allele Frequencies (MAFs) ranging from 0.441 to 0.821 and averaging at 0.62 (Figure 2a). This range and average indicate a substantial presence of genetic variation among the markers. Gene diversity values fluctuated between 0.293 and 0.597, and heterozygosity values varied from 0.016 to 0.719, with overall averages of 0.460 and 0.283, respectively (Figure 2b,c). Furthermore, 28 of the markers boasted PIC values exceeding 0.3, with an overall average PIC of 0.355 (Figure 2d). PIC values are crucial as they measure the informativeness of a marker for genetic studies. The high PIC values, in conjunction with the other genetic diversity indices, confirm the extensive polymorphism and reliability of the candidate core marker set, thereby rendering it highly suitable for fingerprinting analysis and further genetic research. The present set of markers provides a solid foundation for genetic mapping and marker-assisted breeding efforts in eggplant.

**Figure 3:**
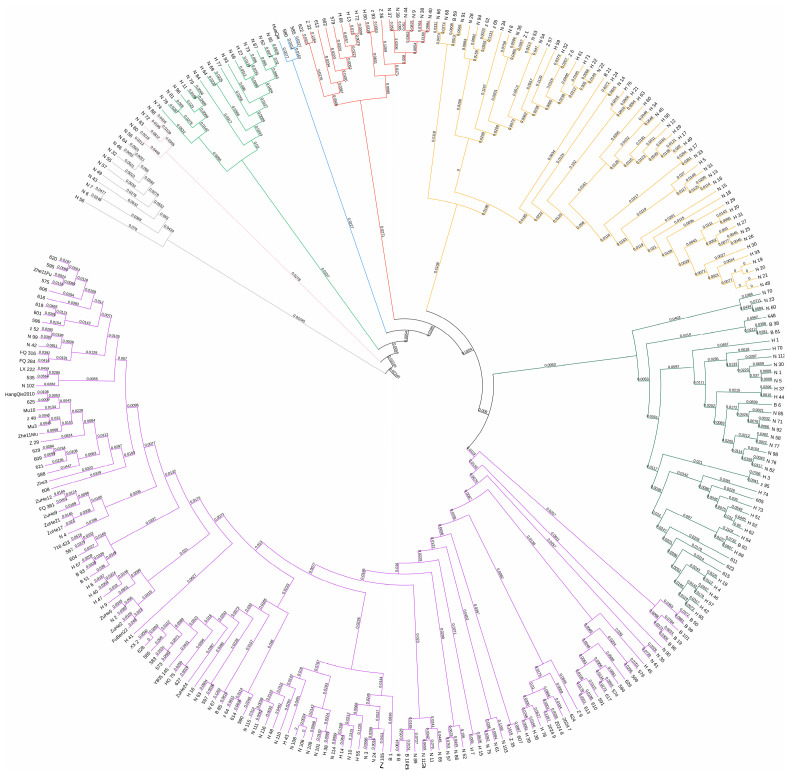
Phylogenetic analyses of 280 eggplant cultivars.

The phylogenetic analysis constructed by the Nei’s 1983 method revealed that the outermost sub-pop 1 consisted of wild eggplant varieties characterized by small green round fruits (Figure 3, Supplementary Table S5). The outermost layer of the phylogenetic tree represents wild types, gradually transitioning inward to cultivated varieties. Sub-pop 2 primarily comprises purple-black eggplants from Europe, while sub-pop 3 contains both striped eggplants and purple-black elongated types. Sub-pop 4 consists of varieties with mixed purple and green fruit exocarps. Sub-pop 5 shows increased diversity in both fruit color and shape. Sub-population 6, primarily originating from the central and northern regions of China, exhibits a diverse range of fruit coloration, encompassing shades of purple-red, purple-black, green, white, and striped varieties. The fruit shapes of this sub-population also display a high degree of diversity, predominantly featuring oblate, spherical, oval, and long oval forms, while also occasionally presenting rare forms such as oxheart and bulbous shapes. The remarkable genetic diversity characteristics of this population provide a precious gene resource pool for analyzing stress resistance mechanisms and carrying out targeted quality improvement research. Its strategic value in the field of crop genetic breeding is worthy of in-depth exploration and utilization. In sub-pop 7, the fruits are generally longer and predominantly purple-black in color, The single phenotype exhibited by this group is likely the result of long-term artificial selection, a phenomenon closely linked to market demands, which further highlights the necessity of molecular marker-assisted breeding. Sub-pop 8 primarily comprised eggplant varieties exhibiting either purple exocarp or long-shaped fruit shapes (or both phenotypes), suggesting that pericarp color and fruit length may have been key traits under human selection. The 128 eggplant varieties within sub-pop 8 were derived from a wide range of sources, spanning countries across Asia, Europe, and the Americas, indicating a narrow genetic base for purple elongated eggplants globally and suggesting a potential risk of genetic erosion.

**Figure 4:**
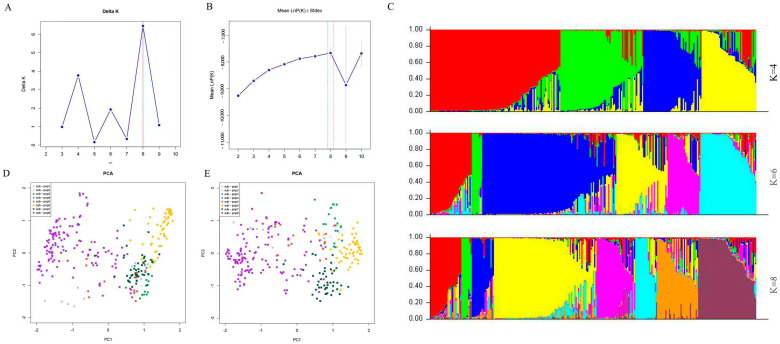
Genetics analysis of 32 candidate core markers in 280 eggplant materials. (**A**) The DK values corresponding to different K measurements. (**B**) The LnP(K) values corresponding to different K measurements. (**C**) Population structure analysis of 280 eggplant cultivars at K = 4, K = 6, and K = 8. (**D**) Principal component analysis by PC1 and PC2. (**E**) Principal component analysis by PC1 and PC3.

Based on the genotyping data of 280 eggplant accessions, population structure analysis was performed using the Structure 2.3.4 software, and the results were further processed via the Structure Harvester website. The analysis indicated that the accessions could be categorized into 4, 6, or 8 groups. This is because these points (K = 4, K = 6, and K = 8) all represent peak inflection points. From K = 2 to K = 8, the natural logarithm likelihood value increased steadily, and almost identical values were seen at K = 8 and K = 10. (Figure 4A–C). To identify the most suitable grouping, the smallest K value associated with the highest ln likelihood was chosen, with K = 8 exhibiting a clear inflection point in the ΔK statistic. This suggests that dividing the eggplant germplasm into eight groups is optimal. This conclusion aligns with the results of the clustering analysis, providing a consistent framework for understanding the genetic diversity and population structure within the eggplant germplasm. The congruity between the Structure software analysis and the clustering analysis validates the findings, reinforcing the reliability of the determined genetic groups. This comprehensive approach to population structure analysis is crucial for genetic studies, as it allows for a better understanding of the genetic relationships among different eggplant varieties and can inform breeding strategies aimed at preserving genetic diversity and improving crop characteristics.

In order to further observe the classification of the population, the 280 eggplants’ typing results were used for PCA analysis (Figure 4D,E). The results revealed that, after dividing the 280 accessions into eight sub-pops, sub-pop 1 exhibited a distant genetic relationship from the rest, while sub-pops 2–5 were closely related and difficult to distinguish. Sub-pops 6, 7, and 8 appeared more independent, sub-pop 6 is predominantly derived from central and northern China, characterized by highly diverse fruit colors and shapes (such as purplish-red, green, white, spherical, and oval). This diversity likely stems from long-term natural hybridization and local variety domestication in the region, leading to the accumulation of unique SNP combinations in the genome. In sub-pop 7, fruits are generally longer and predominantly purple-black, presumably due to the artificial selection that has formed unique genotypes, increasing the genetic distance from other subgroups. Sub-pop 8 mainly consists of eggplant varieties with purple pericarp or long fruit shape (or both phenotypes), and it is speculated that they may have a common ancestral origin. All three subgroups exhibit independence in PCA analysis, corresponding to distinct genetic clustering characteristics, which further confirms the uniqueness of their genetic backgrounds and differences in evolutionary pathways. By integrating the results of the phylogenetic tree and PCA analysis, we further confirmed the stable classification of wild species and long purple exocarp varieties. In contrast, semi-wild and other cultivated species exhibited intricate genetic transitions.

The combined results of phylogenetic analysis, population structure analysis, and PCA analysis mutually reinforce each other, enhancing the reliability of this study and providing a solid scientific basis for the construction of eggplant fingerprint maps and the identification of core germplasm resources.

### 2.4. Construction of SNP Fingerprints

**Figure 5:**
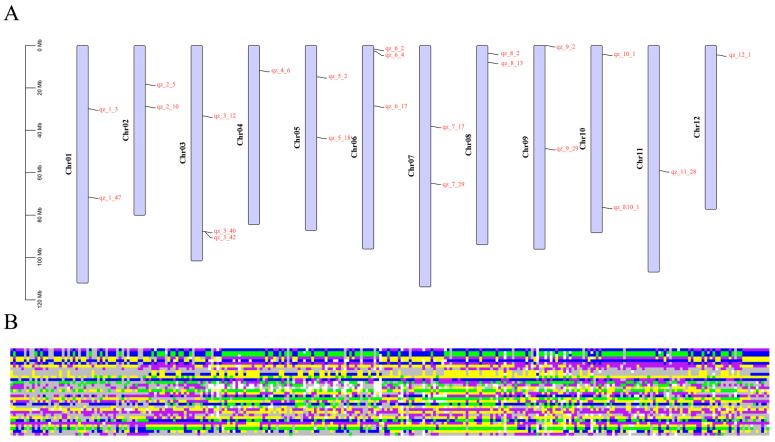
Distribution of 23 core markers and fingerprinting. (**A**) Distribution of 23 core markers on 12 chromosomes. (**B**) Fingerprints of the 280 eggplant materials. Each row represents a genome and each column represents a sample. Pure genotypes are AA = yellow, CC = green, GG = blue, TT = purple; heterozygous genotypes are gray; no call genotypes are NA = white.

The minimum core markers were determined using the SNPT 0.3 software, which calculated that a set of 23 KASP markers constituted the most efficient core marker set for genetic analysis (Figure 5A, Supplementary Table S6). When this set was applied to 280 eggplant samples, it was found that 10 samples could not be accurately identified due to the challenges in differentiating certain marker results. This phenomenon may be caused by the following reasons: firstly, the genotypes of the samples are highly similar, making it difficult to effectively distinguish them; secondly, the limited number of markers may affect the identification effect; and finally, human operational steps (such as incorrect labeling, confusion in packaging, data recording errors, etc.) may also lead to the incorrect identification of the sample identities. Additionally, four samples (N_19, N_20, N_21, and N_48) exhibited identical genotyping results, as did N_106 and N_108, as well as 565 and 628, which was consistent with the clustering analysis results. N_19, N_20, N_21, and N_48 are extremely similar, all being green oval eggplants, with the first three originating from Henan. N_106, N_108, 565, and 628 are purple-black long eggplants, with the first two from Japan. These similar genetic backgrounds may lead to difficulties in distinguishing their genotyping results. To facilitate the rapid identification of eggplant varieties, we integrated the genotyping results of 32 KASP core markers and 280 samples to construct a fingerprint by R (Figure 5B).

### 2.5. Construction of Core Germplasm

Using the CoreHunter R package, we conducted an analysis on the core germplasm resources and established a representative eggplant core collection consisting of 56 accessions (Supplementary Table S7), which represents approximately 20% of the total germplasm resources. The collection includes samples from 19 provinces and 9 countries across 3 continents, covering 3 main colors (purple, green, white) and various fruit shapes. The CoreHunter analysis was instrumental in calculating key genetic diversity indices, which are crucial for assessing the quality and representativeness of the core collection. The Rogers’ genetic distance (MR) and Cavalli–Edwards genetic distance (CE) both measured 0.36, indicating a moderate level of genetic differentiation among the accessions. The Shannon diversity index (SH) was 3.41, expected heterozygosity (HE) was 0.61, and the PIC value was 0.74, all of which suggest a high level of genetic diversity within the collection. The allele coverage (CV) reached 100% (Table 1). Overall, based on the genetic diversity data, the core collection exhibited notable diversity characteristics.

## 3. Discussion

China is a major producer and consumer of eggplant, with various types cultivated and traded nationwide. The elongated purple variety of eggplants is the preferred choice among residents in the eastern coastal regions of China and dominates the markets in Zhejiang and surrounding provinces. Consequently, we prioritized the collection of purple elongated eggplant germplasm to support identification and breeding efforts. Currently, the genetic diversity of purple elongated eggplants is relatively limited, making the introduction of new germplasm essential for breeding progress. However, the extensive range of germplasm sources often leads to the same material being known by different names across regions or different materials sharing the same name. This creates significant challenges for breeders, as it is difficult to distinguish or consistently name these materials based solely on physical traits. Thus, establishing a reliable germplasm identification system is crucial. Current research on fingerprint construction and genetic diversity analysis in purple elongated eggplants is relatively limited. Julio E. Muñoz-Falcón et al. studied the renowned Spanish eggplant variety *Listada de Gandía* [35]. In total, 17 SSR markers and 32 EST-SSR markers were used to assess the genetic diversity and relatedness among 42 eggplant samples and to construct a fingerprint. This study identified both universal markers and markers specific to *Listada de Gandía*, contributing to the preservation of local varieties and the protection of breeders’ rights. By employing an enriched genomic library, a set of 55 SSR markers was developed to effectively discriminate distinct eggplant varieties originating from diverse geographical regions and exhibiting different morphologies [36]. This differentiation classified eggplant origins into two major categories: Eastern (eastern and southeastern Asia) and Western (Mediterranean basin, Central Europe, Africa, and America). The findings support efforts in conservation, management, and breeding research for eggplant varieties and provide new insights into their genetic backgrounds. Using the eggplant genome 67/3 V3 [37] and resequencing data from 45 unique lines, a targeted SNP-seq approach identified 219 SNPs for genotyping 377 eggplant varieties, leading to the construction of a detailed fingerprint [38]. Population structure analysis and PCA identified three distinct population clusters, indicating that selective breeding has significantly impacted eggplant fruit shape. This has resulted in narrow genetic backgrounds in varieties with round and oval fruits, highlighting a risk of genetic erosion for these shapes. Additionally, a Perl-based method [39] selected 36 core SNPs capable of distinguishing 95% of eggplant varieties.

In this study, 224 high-quality SNPs were identified by the resequencing data from 49 materials and 96 selected SNPs were typed by KASP technology. Various genetic analyses demonstrated that the genotyping results of 280 eggplant samples were robust, and the KASP markers were reliable. KASP is renowned for its high cost-effectiveness, simple operation, and high specificity, and has become a mainstream genotyping technology in biological genetics research. This technique effectively overcomes the limitations of traditional methods (such as gel electrophoresis and Sanger sequencing) in terms of throughput, cost, and precision, enabling rapid and high-throughput detection of single nucleotide polymorphisms (SNPs) and insertions/deletions (Indels), thus providing an efficient tool for genetic analysis. Meanwhile, in this study, the inclusion of a larger and more geographically diverse sample population has effectively improved the understanding of global eggplant genetic diversity. The 280 eggplants were consistently grouped into 8 clusters through phylogenetic analysis, population structure analysis, and PCA. The observed groupings exhibited a correlation with the origins and phenotypic characteristics of the samples, as eggplant varieties sharing common origins and displaying similar phenotypes tended to cluster together. Purple elongated eggplant varieties primarily cluster within subgroup 8, which is primarily composed of eggplants with purple skin or elongated fruit. The wide sourcing of these eggplants across three continents suggests a global similarity in purple elongated eggplant germplasm, likely attributed to extensive human selection over time, and resulting in a narrowed genetic background. Consequently, purple elongated eggplant varieties are highly susceptible to genetic erosion. In breeding programs, it is essential to consider the risks associated with close genetic relationships, such as reduced potential for trait improvement, increased genetic vulnerability, and inbreeding depression. An SNP fingerprint for the 280 eggplant germplasms was developed using 23 core KASP markers, and a core collection comprising 56 germplasm samples was established, effectively capturing the allelic diversity of the original 280 materials. The results indicate that the SNP-based DNA fingerprint is reliable and accurate, providing technical support and a theoretical basis for eggplant variety identification, kinship analysis, and germplasm collection and conservation, thereby promoting breeding development. Compared to previous studies, this research establishes a foundation for constructing an eggplant fingerprint using KASP technology, with a particular focus on analyzing the genetic diversity of elongated fruit eggplant varieties. The findings indicate a risk of genetic diversity loss within purple elongated eggplant types, underscoring the need to broaden the selection of genetic materials in breeding programs.

A researcher has systematically expounded on the strategies for improving crop salt tolerance from both the genetic mechanism and breeding technology perspectives. They particularly emphasized that by exploring genetic diversity (such as developing the stress-resistant gene resources of crop wild relatives) and integrating modern biotechnologies (such as gene editing and intelligent breeding systems), the bottlenecks in traditional breeding for salt tolerance improvement can be overcome [10]. This discussion provides important inspiration for this research. Although this study did not conduct in-depth analysis of the salt tolerance of eggplants, the SNP markers developed based on the KASP technology can provide key technical support for quickly and accurately locating target trait genes (such as salt tolerance-related genes). Combined with the conclusion in the literature that “wild cultivated materials contain abundant stress-resistant genes”, we can further promote the breeding utilization, domestication improvement, and innovative practice of germplasm resources through the exploration and identification of genetic backgrounds.

Nonetheless, the scope of material collection and the relatively limited number of developed KASP markers impose certain constraints on genetic analysis. Phylogenetic clustering and PCA revealed that a few materials exhibited close kinship, making them challenging to differentiate, with occasional clustering inaccuracies, such as the grouping of H_56 within the wild-type cluster. With the rapid advancement of DNA sequencing and genotyping technologies, the limited marker quantity, stemming from the adoption of the principle of minimum markers for maximum sample discrimination, meant that the genotyping strategy focused on distinguishing the largest number of samples with the fewest markers, which likely led to insufficient marker coverage to capture the unique genetic signatures of certain samples; additionally, potential sample information errors arising from human operational mistakes in handling the large sample volume cannot be ignored. DNA fingerprinting has entered a new era. Early technologies, constrained by high costs and technical limitations, relied on a limited number of markers to differentiate germplasm materials. However, with the maturity of high-throughput sequencing and chip technologies, experimental costs have significantly decreased, and the number of markers is no longer a limiting factor. High-density, multi-site gene chips allow for the rapid screening of numerous samples, greatly enhancing the efficiency and accuracy of germplasm identification. Currently, a single gene chip can detect tens of thousands of genetic loci [40,41], facilitating large-scale testing and providing robust technical support for germplasm innovation and breeding selection. In the future, as technology continues to evolve, DNA fingerprinting is poised to have broader applications in agricultural genetic improvement and variety protection. It will not only provide researchers with comprehensive datasets but also facilitate the precise management and optimal utilization of global agricultural resources.

## 4. Materials and Methods

### 4.1. Plant Materials and DNA Extraction

The present study focuses on the usage of 286 eggplant accessions for constructing an eggplant fingerprint. Among these, 174 eggplant cultivars were provided by the Zhejiang Academy of Agricultural Sciences (ZAASs), while an additional 112 samples were obtained from the Chinese Academy of Agricultural Sciences (CAASs). Resequencing data from 49 representative eggplant accessions were used for high-quality SNP identification, with the data from 4 of these already published [42]. An additional 286 eggplant accessions were subjected to fingerprinting analysis, with the exclusion of the 49 representative materials that underwent resequencing. The DNA of eggplant cultivars was extracted from young leaf tissues using a modified CTAB method. DNA quality and quantity were determined by a NanoDrop 2000 Spectrophotometer (Thermo Fisher Scientific, Waltham, MA, USA).

### 4.2. SNPs Identification

The resequencing of 45 accessions was completed by Novogene Co., Ltd. (Illumina, San Diego, CA, USA) Initially (stored in CNGBdb under the accession number CNP0006759), 4 accessions have been published, and a total of 49 representative accessions were subjected to resequencing data. Raw data quality control was performed on the resequencing data using fastaqc, followed by the removal of low-quality sequences, and adapters were removed using fastp v0.23.4 software [43]. The BWA v0.7.17 (MEM) software was used to compare with the HQ1315 genome [44], and the GATK v4.5.0.0 software was employed for SNP calling, including base quality score recalibration, indel realignment, and variant filtering to obtain the SNP set. The set of SNPs was filtered by using the following criteria: (1) Python 3.9 scripts were used to extract sequences that are 100 bp upstream and downstream of the identified SNPs, ensuring that these regions are devoid of any other variant locations, not only SNPs. (2) GATK software was used for rough filtering of SNPs with parameters QD < 2.0, MQ < 40.0, MQRankSum < −12.5, and ReadPosRankSum < −8.0 [45] (3) Then, VCFtools 4.3 software, designed for processing VCF (Variant Call Format) files, was utilized to perform additional filtering based on standard parameter settings [46]. According to the filtering criteria, we adjusted some parameter settings, as follows: —mac 3—maf 0.05—max angles 2—min angles 2—mean DP 4—minQ 30—max missing 0.5. Finally, 224 high-quality SNPs were obtained for subsequent experiments.

### 4.3. KASP Development and Genotyping

Sequences 100 bp upstream and downstream of the candidate SNPs were extracted for the development of KASP markers. The design of two allele-specific primers (FAM tail: 5′-GAAGGTGACCAAGTTCATGCT-3′, HEX tail: 5′-GAAGGTCGGAGTCAACGGATT-3′) and one common primer was performed for each KASP marker. The parameters for KASP marker development are as follows: GC% is less than 60%, the product size should not exceed 120 bp, and the annealing temperature should be within the range of 55 to 62 °C. The primers were synthesized by Zhejiang Yihe Genetics Co. Ltd (Hangzhou, China). The KASP genotyping assay was performed in a 1.6 mL PCR reaction mixture, which contained 0.8 mL of KASP Master Mix (LGC, Biosearch Technologies, Hoddesdon, UK), about 0.05 mL of primers, and 0.8 mL of DNA with a concentration of 10–20 ng/mL. The PCR conditions included a step of 94 °C for 15 min, followed by 10 touchdown cycles at 94 °C for 20 s and 61–55 °C for 60 s, with a gradual decrease in temperature of 0.6 °C per cycle. This was then followed by 26 cycles of 94 °C for 20 s and 55 °C for 60 s. Subsequently, fluorescence signal acquisition and analysis of PCR products were conducted using the IntelliQube system (LGC, Biosearch Technologies), generating schematic diagrams of KASP marker genotyping results for use in subsequent research.

### 4.4. Population Differentiation and Genetic Diversity Indices

The KASP results were organized by Excel 2016. Genetic diversity was calculated by PowerMarker V3.25. A two-by-two genetic distance matrix was generated from the genotype data, which served as the basis for constructing a neighbor-joining (NJ) tree by using Nei’s 1983 standard genetic distance. The NJ tree was visualized with MEGA-X and iTOL (https://itol.embl.de/ (accessed on 30 May 2020)). The phylogenetic analysis was conducted by Structure 2.3.4. The parameters were set as follows: burn-in period of 10,000 iterations, followed by 100,000 MCMC repetitions, and the DK method (DK: 2–10) was used to determine the optimal number of groups (k), with 10 iterations. After running the software, the resulting files were compressed and uploaded to the online tool Structure Harvester (https://lmme.ac.cn/StructureSelector/index.html ( accessed on 30 May 2020)) to determine the optimal number of clusters based on the method described by Evanno et al [47]. Principal component analysis (PCA) was performed using Tassel 5.1, and visualization was conducted by R.

## 5. Conclusions

In this study, we established a robust DNA fingerprinting system for eggplant germplasm using KASP technology, providing a powerful tool for genetic diversity analysis and cultivar identification. Through resequencing of 49 representative accessions, we identified 224 high-quality SNPs, of which 96 were developed into KASP markers. After rigorous validation in 286 samples, 32 core KASP markers were selected, demonstrating high polymorphism with an average PIC value of 0.36 and gene diversity (GD) of 0.46. These markers enabled the successful classification of 280 eggplant accessions into 8 distinct genetic clusters through phylogenetic tree, population structure (Structure 2.3.4), and principal component analysis, with clustering strongly correlated with geographic origins and phenotypic traits (e.g., fruit color and shape). Through further analysis, a minimum core marker set consisting of 23 SNPs was selected. This marker set is capable of distinguishing almost all the tested genetic resources, while a core germplasm subset of 56 accessions was established to capture the genetic diversity of the entire collection. The core germplasm exhibited high genetic representativeness, with a Shannon diversity index (SH) of 3.41 and allele coverage (CV) of 100%, providing a valuable resource for efficient germplasm management and breeding. Our findings highlight the utility of KASP-based fingerprinting in resolving genetic relationships and addressing misnaming issues in eggplant germplasm. The identified markers and core germplasm not only facilitate accurate cultivar identification and protection of breeder rights but also accelerate marker-assisted selection (MAS) for trait improvement, such as enhancing resistance and nutritional quality. However, challenges remain in capturing rare genotypes and improving marker density for fine-scale genetic analysis. Overall, this study establishes a foundational framework for eggplant genetic research and breeding, emphasizing the importance of integrating high-throughput molecular markers with germplasm conservation strategies. The resources and methodologies developed here will contribute to sustainable eggplant improvement by enabling precise genetic characterization and targeted utilization of genetic diversity.

## Figures and Tables

**Table 1:** Diversity analysis of core germplasm.

Original Germplasm	Core Germplasm	MR	CE	SH	HE	NE	CV
280	56	0.36	0.36	3.41	0.61	1.00	100%

## Data Availability

The original data generated during the study are available from the corresponding author Qingzhen Wei (weiqz@zaas.ac.cn) upon reasonable request.

## Supplementary Materials

The following supporting information can be downloaded at: https://www.mdpi.com/article/10.3390/ijms26115312/s1.
