# Supplementary material for: Construction of SNP Fingerprinting and Genetic Diversity Analysis of Eggplant Based on KASP Technology
Source: Int J Mol Sci. 2025 May 31;26(11):5312. doi: 10.3390/ijms26115312 (PMC12154498; doi:10.3390/ijms26115312)
Supplement: Supplementary file 1 [file ijms-26-05312-s001.zip › Supplementary Figure.pdf]

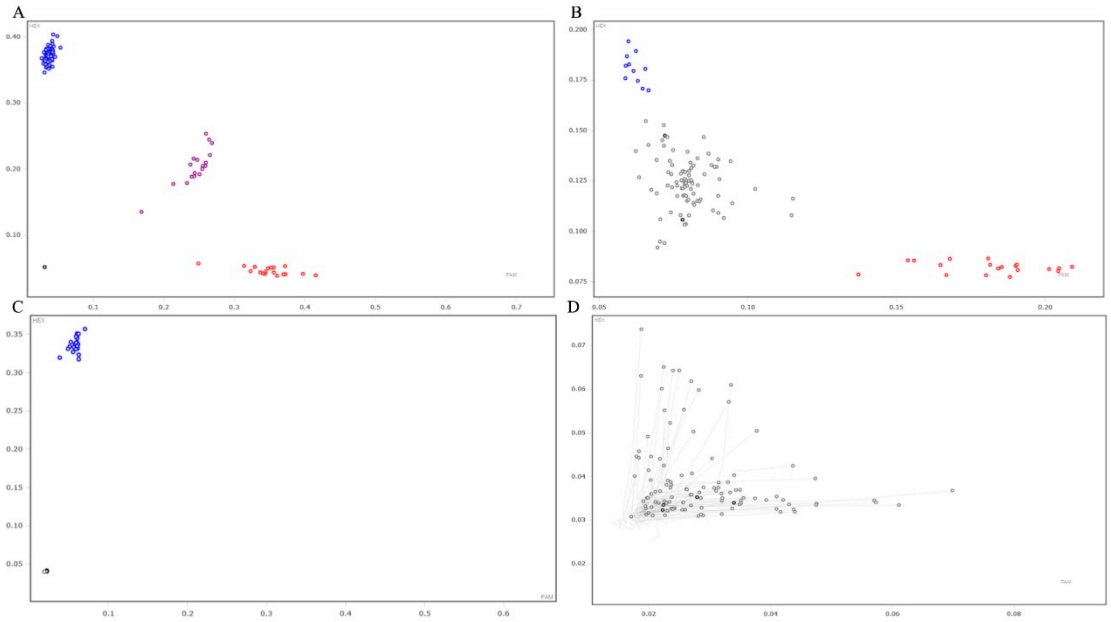

Supplementary Figure S1

Four representative KASP assay results. (A) A KASP marker with good typing results. (B) A difficult to classify KASP marker. (C) A KASP marker with monomorphism. (D) A KASP marker with no call.
